# Selection of the Inducer for the Differentiation of Chicken Embryonic Stem Cells into Male Germ Cells *In Vitro*

**DOI:** 10.1371/journal.pone.0164664

**Published:** 2016-10-14

**Authors:** Yani Zhang, Yingjie Wang, Qisheng Zuo, Xiaoyan Wang, Dong Li, Beibei Tang, Bichun Li

**Affiliations:** 1 College of Animal Science and Technology, Yangzhou University, Yangzhou 225009, Jiangsu province, China; 2 Key Laboratory for Animal Genetics, Breeding, Reproduction, and Molecular Design of Jiangsu Province, Yangzhou, 225009, Jiangsu province, China; Qingdao Agricultural University, CHINA

## Abstract

Several inducers have been used to differentiate embryonic stem cells (ESCs) into male germ cells but the induction process has been inefficient. To solve the problem of low efficiency of inducer for ESCs differentiation into male germ cells, all-trans retinoic acid (ATRA), Am80(the retinoic acid receptor agonist), and estradiol (E2) was used to induce ESCs to differentiate into male germ cells *in vitro*. ESCs were cultured in media containing ATRA, Am80, or E2 respectively which can differentiate ESCs into a germ cell lineage. In process of ATRA and Am80 induction Group, germ cell-like cells can be observed in 10 days; but have no in E2 induction Group. The marker genes of germ cell: *Dazl*, *Stra8*, *C-kit*, *Cvh*, *integrinα6*, and *integrinβ1* all showed a significant up-regulation in the expression level. The ATRA-induction group showed high expression of *C-kit* and *Cvh* around 4 days, and integrinα6 and integrinβ1 were activated on day 10, respectively, while the E2-,Am80- induction group showed a high expression of *C-kit* as early as 4 days immunocytochemistry results shown that, *integrinα6* and *integrinβ1* could be detected in the ATRA-, Am80-, and E2-induction group, Positive clones in the ATRA group were greater in number than those in the other two groups. we conclued that ATRA, Am80, and E2 can promote the expression of the corresponding genes of germ cells, and had different effect on the differentiation of ESCs into male germ cells. ATRA was the most effective inducer of germ cell differentiation.

## Introduction

Embryonic stem cells (ESCs), which derive from the inner cell mass, can differentiate into almost all types of cells because of their pluripotency. As an ideal cell model, ESCs are often used to explore cell development and differentiation, especially for germ cell development[1]. ESCs have the potential to be induced to differentiate into male germ cells in vitro. Much research has focused on the differentiation of ESCs into male germ cells[2–4]. In these studies, the inducers varied, and differentiation into germ-like cells was based on the expression of specific germ cell genes. However, the induction process is inefficient and unpredictable and the in vitro expression pattern of genes related to male reproductive cells derived from ESCs was not consistent with expression in vivo. Retinoic acid (RA), a fat-soluble, small molecule metabolite of vitamin A, is the most commonly used inducer of differentiation of ESCs into male germ cells in vitro[5–7]. All-trans retinoic acid (ATRA) is the main form. Retinoic acid has an important role in the development and maintenance of the normal physiological state, such as mediation of cell differentiation, proliferation, apoptosis, and regulation of immune function[8,9]. In addition, ATRA is necessary during the process of spermatogenesis in vivo[8]. ATRA can induce the expression of many genes, such as *Stra8*, *Sycp1*, and *Dazl*, and promote the differentiation of mouse ESCs into male germ cells[10]. ATRA can promote primitive germ cell survival and proliferation and maintain high mitotic ability, and can also induce mouse ESCs to differentiate into primitive germ cells[11]. Tamibarotene (Am80) is a new retinoic acid analog and a specific agonist for the retinoic acid receptor alpha[12,13]. Its action is similar to retinoic acid, but there are few reports about its role in the process of male germ cell differentiation in vitro[14].

Estradiol (E2) is known to have a key role in the process of gonad differentiation. Female and male chicken embryos express estrogen receptor (ER) alpha in the gonads after 3.5 to 4.5 days of incubation[15]. Exogenous E2 (17-beta estradiol) can significantly promote proliferation of ovarian germ cells from chicken embryos after treatment for 48 hours[16–18], probably by promoting the synthesis of estrogen or its receptor.

Poultry is an important animal model for studying developmental biology. It is convenient to acquire ESCs from the chicken embryos, and there are no limits on material and ethical considerations. Our group has been committed to the use of domestic chicken ESCs to study directional differentiation in vitro[19] and the induction of ESCs into male reproductive cells by a specific induction system. However, the efficiency of induction is very low. In order to improve the efficiency of induction and further optimize the induction system, ATRA, Am80 and E2 were used in this study to induce ESC differentiation into male germ cells in vitro.

## Materials and Methods

### Ethics statement

The Rugao yellow chickens used in this study were provided by the Institute of Poultry Science of the Chinese Academy of Agriculture Sciences. Procedures involving the care and use of animals conformed to the U.S. National Institutes of Health guidelines (NIH Pub. No. 85–23, revised 1996) and were approved by the Yangzhou University Institutional Animal Care and Use Committee.

### Animal Materials

Fertilized eggs were from the Rugao Huang chicken from the Poultry Institute, Chinese Academy of Agricultural Sciences. The eggs were selected for the isolation of blastoderm cells within 5 hours of being laid[20]. The animal-use protocol was approved by the laboratory-animal management and experimental-animal ethics committee of Yanzhou University.

### Isolation, culture and sex determination of chicken ESCs

Fresh fertile eggs were sterilized by bromogeramine followed by 75% ethyl alcohol. The egg blunt was then broken and the egg white removed. The blastoderm in the yolk was isolated by scissors, taken out by medicinal ladle, and washed with PBS 2–3 times. The yolk membrane and yolk around the blastoderm was wiped off. Pure blastoderm was centrifuged for 6 minutes at 1000xg, dispersed with phosphate buffered saline (PBS), and adjusted to a cell density of 1×10^6^/mL.

Blastoderm cell suspensions (50 μL) were used to extract DNA according to the instructions of the MicroElute Genomic DNA kit (Omega Bio-tek, Inc., Norcross, GA, USA). The concentration of DNA was adjusted to 50 μg/mL and the *CHD-W* gene was amplified by PCR. The forward primer was GTTACTGATTCGTCTACGAGA and the reverse primer was ATTGAAATGATCCAGTGCTTG. The 10 μL PCR amplification system contained 1 μL 10×PCR buffer‚2.5 μmol dNTP Mixture,2 μmol of each primer, 2.5 U Taq, and 0.1 μg DNA. The PCR amplification procedure began with pre-denaturing at 94°C for 5 min, then a run for 30 cycles including denaturing at 94°C for 50 s, annealing at 49°C for 50 s, extending at 72°C for 30 s, extending at 72°C for 10 min, and finally preserving at 4°C. The blastoderms of ZZ genotype were selected for the following culture and induction. ESCs culture medium contained 10% fetal bovine serum (FBS), 2% chicken serum, 5.5×10^−5^ mol/L β-mercaptoethanol, 2 mM L-glutamine, 1% non-essential amino acid, 0.1 ng/mL leukemia inhibitor factor, 5 ng/mL human stem cells factor, and 10 ng/mL basic fibroblast growth factor (Sigma) in DMEM.

### Differentiation of chicken ESCs *In Vitro*

ESCs of the second generation were passaged at 10^−5^ cells/well into 24-well plates using Sertoli cells as a feed layer. ATRA (10^−5^ mol/L), Am80 (10^−6^ mol/L), and E2 (1 μg/mL) were added to the culture medium respectively to induce male chicken ESC differentiation into germ cells. The control group used basal culture medium. Each group had three repetitions. Every two days, the cells were observed by inversion microscope and were collected for RNA extraction and immunocytochemical detection.

### Quantitative Real-Time PCR (qRT-PCR)

The cells were collected at 0, 4(PGC-like cell appear *in vitro*), 10(SSC-like cell appear *in vitro*)days and RNA was extracted using the RNeasy kit (Qiagen) and reverse transcribed to cDNA. The expression of specific germ cell genes was evaluated by real-time PCR using the 7500 System (Applied Biosystems, Grand Island, NY, USA) in a total volume of 20 μl containing SYBR mix according to the manufacturer’s instructions (SYBR Premix Ex Taq^™^, Takara Biotechnology, Co.). Chicken β-actin was used as an internal reference to normalize relative gene expression.

The gene expression was measured using the 2^− ΔΔCt^ method. All PCR products were run on ethidium bromide-stained agarose gels and confirmed using melting curve analyses to assess product quality, The prime of the genes were supplied in Table 1.

**Table 1:** Primer information for qRT-PCR.

Gene	Primers for qRT-PCR	Tm(°C)	Size(bp)
*Nanog*	F: TGGTTTCAGAACCAACGAATGAAG	64	180
	R: TGCACTGGTCACAGCCTGAAG		
*Sox2*	F: GAAGATGCACAACTCGGAGATCAG	64	100
	R: GAGCCGTTTGGCTTCGTCA		
*C-kit*	F: GCGAACTTCACCTTACCCGATTA	64	150
	R: TGTCATTGCCGAGCATATCCA CATATCCTTGGCAGGTTGTTGA		
*Cvh*	F: TGTCTTGAAGGCCTCGTTTG	61	138
	R: CATATCCTTGGCAGGTTGTTGA		
*integrinα6*	F: TGTTTGTGGGGACCAGATTG	53	120
	R: CCAGGTGACATTTCCCATCA		
*integrinβ1*	F: GAAACCCGGGATATCATTGG	53	140
	R: CAGCAACACCTTGCTGACAG		
*Dazl*	F: TGTCTTGAAGGCCTCGTTTG	61	138
	R: CATATCCTTGGCAGGTTGTTGA		
*Stra8*	F: CCACGGCTATTTCACACCTCTG	64	114
	R: GCTCTTGGCAAGCATCCGTA		
*β-actin*	F: CAGCCATCTTTCTTGGGTAT	60	164
	R: CTGTGATCTCCTTCTGCATCC		

### Immunocytochemical detection

At the end of the differentiation period, the cells of each group were fixed with 4% paraformaldehyde for 30 min. Then, the cells were washed with 3x PBS. Next, the cells were blocked with 10% bovine serum albumin (BSA)-PBS at 37°C for 30 min and specific antigen (1:200 anti-Nanog, 1:200 anti-SSEA-1, 1:200 anti-Cvh, 1:250 anti-C-kit, 1:200 integrin α6, and 1:200 integrin β1; Millipore, Inc., Billerica, MA, USA) for one night at -4°C. Following a wash with 3x Phosphate Buffered Saline Tween (PBST), fluorescein isothiocyanate (FITC)- or PE-labeled goat anti-mouse immunoglobulin M (Bioss, China) was added and the incubation was continued at 37°C for 2 hours. The cells were then incubated with DAPI staining solution for 3 min at 37°C. After washing again with PBST, cells were observed under an inverted fluorescence microscope.

### FACS

Cell suspension induced of 10 days were Collect in 1.5 mL centrifuge tube, 1000 g centrifugal for 5 min, abandon the supernatant, add the integrin α6(1:200) antibody, 4°C for 1 to 2 h incubation, centrifugal supernatant. Precooling PBS centrifugal washing twice. Add precooling PBS 500 uL for FACS analysis

## Results

### Identification of chicken ESCs

**Fig 1:**
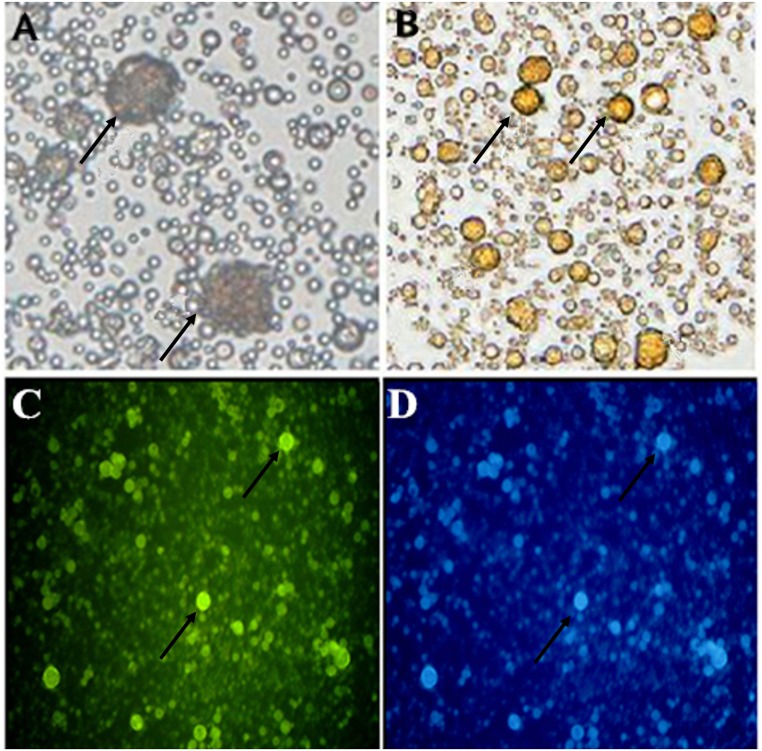
Pluripotent identification of the 3rd generation of chicken ESCs (400x). A: Cultured ESCs with clones forming a “nest”; B: ESCs stained by AKP. Pluripotent ESCs are stained brown; C: ESCs stained for SSEA-1. Pluripotent ESCs are stained green; D: ESCs stained with DAPI;

Undifferentiated ESCs can form obvious clones when cultured in vitro (Fig 1A). The cell clones, which resembled a bird nest, had a clear boundary. The cell clones were positive for alkaline phosphatase (AKP) staining and showed brown in the photos (Fig 1B). ESCs were positive for the expression of stage-specific embryonic antigen SSEA1(Fig 1C and 1D).These results show that ESCs isolated and cultured in vitro are undifferentiated.

### Cell morphological changes after treatment with different inducers

**Fig 2:**
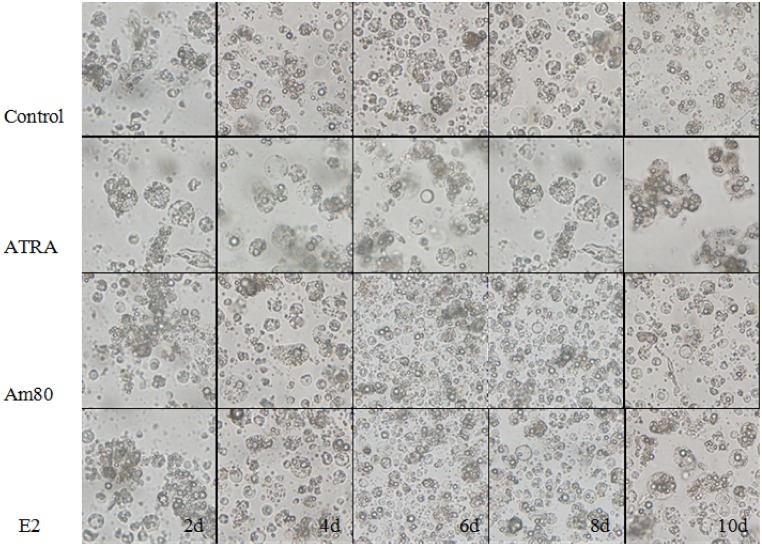
The morphological changes in control cells and cells treated with differentiation inducers (400×). The ATRA-treated group showed few embryoid bodies (EB) on day 2 and the EB number increased during days 4–10. Large numbers of SSC-like cells were observed on day 10. The Am80-treated group showed EB around 4 days, and SSC-like cells were observed on day 10. The E2-treated group showed EB at day 4 and the EB number increased during days 4–8. SSC-like cells were observed around day 10;

The cellular morphology was different in the groups treated with different inducers (Fig 2). The cells in the control group proliferated more slowly. Only a small number of cell colonies had formed after 2–4 days in culture and had begun to differentiate at 6–10 days. In the ATRA-induced group, embryoid bodies gradually formed after 2–4 days of culturing and the numbers increased. At 6–8 days, the embryoid bodies began to break down. Germ-like cells appeared and grew close together, resembling grape bunch-like shapes at 10 days. In the Am80 induction group, embryoid bodies began to appear and gradually increased after 4–6 days of culturing. At 8–10 days, embryoid bodies disintegrated into small, round cells but have no germ-like cells. The cells changes in the E2-induced group were similar to those in the Am80-induced group.All the results shown that ATRA, Am80, and E2 had different effect on the differentiation of ESCs into male germ cells.

### Specific gene expression in each group measured by qRT-PCR

**Fig 3:**
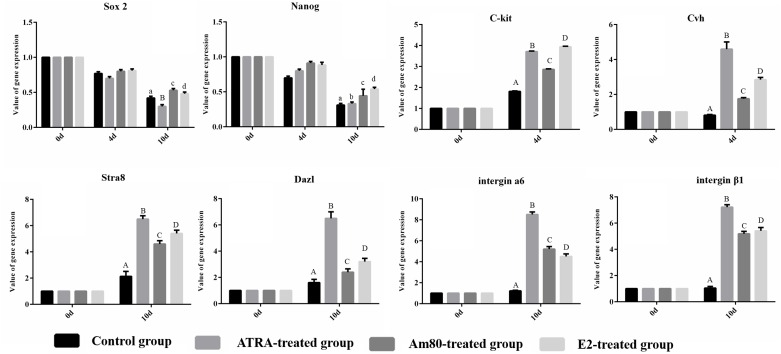
Related gene expression under different inducers measured by qRT-PCR. The ATRA-treated group showed high expression of *C-kit* and *Cvh* around 6 days, and *integrin α6* and *integrin β1* on days 8 and 10, respectively. The Am80-treated group showed high expression of *C-kit* around day 8, and *Cvh* on day 10, while *integrin α6* and *integrinβ1* both showed high expression until day 10. The E2-treated group showed high expression of C-kit as early as day 4, and integrin α6 and integrinβ1 showed high expression until day 10. A: Control group; B: ATRA-treated group; C: Am80-treated group; D: E2-treated group.

We tested the expression of genes specific to stem cells and germ cells at days 0, 4 and 10d of the induction period in each group (Fig 3, S1 Table). We measured the ESC-specific genes *Nanog* and *Sox2*, the primordial germ cell (PGC)-specific genes *C-kit* and *Cvh*, and the SSC-specific genes *Dazl*, *Stra8*, *integrin α6* and *integrin β1*. In the control group, the expression of *Nanog* and *Sox2* increased at 0–4 days and then decreased in the 10th day of induction. The expression of *C-kit*, *Cvh*, *Dazl*, *Stra8*, *integrin α6*, and *integrinβ1* did not change significantly in the control group. It is possible that the cells began to differentiate when cultured in vitro without the inducers and cytokines, such as LIF, GDNF, etc. In the three induction groups, the expression of *Nanog* and *Sox2* decreased over time in culture in a manner similar among the three groups. The expression of the germ cell-specific genes *C-kit*, *Cvh*, *Dazl*, *Stra8*, *integrin α6*, and *integrin β1* increased at different time points among the three groups during the induction period. These results suggest that the three inducers can induce ESCs to differentiate into germ-like cells.All the results shown that ATRA, Am80, and E2 can promote the expression of the corresponding genes of germ cells *in vitro*.

### Detection of cell chemoimmunology

**Fig 4:**
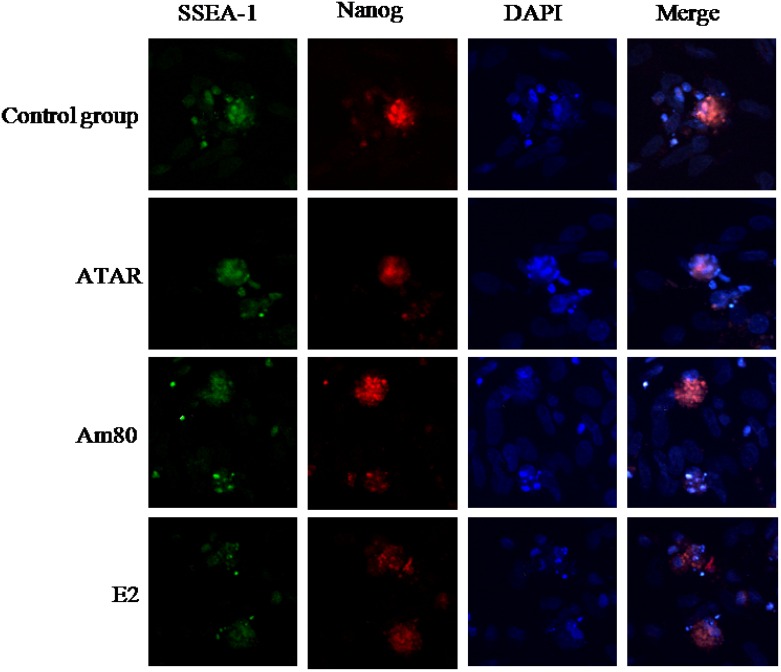
Expression of proteins in EBs as detected by immunofluorescence after 4 days of induction (400×). Immunofluorescence showed EBs that were positive for *SSEA-1* (green) and *Nanog* (red).

**Fig 5:**
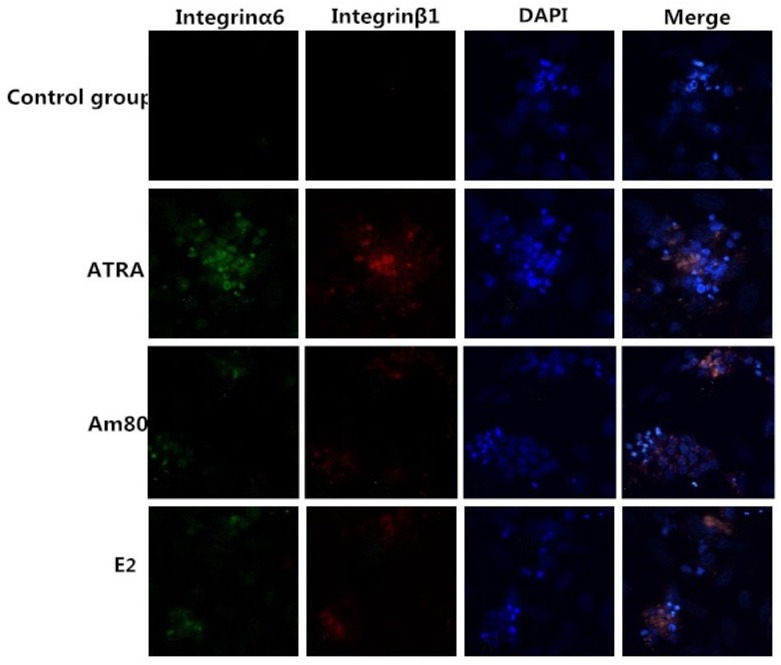
Expression of proteins detected by immunofluorescence (400×). Indirect immunofluorescence staining results of the SSC-specific proteins integrin α6 (green) and integrin β1 (red), and DAPI staining (blue), respectively. After 10 days of induction, the ATRA-, Am80-, and E2-treated groups all contained SSC-like cells while the control group did not.

**Fig 6:**
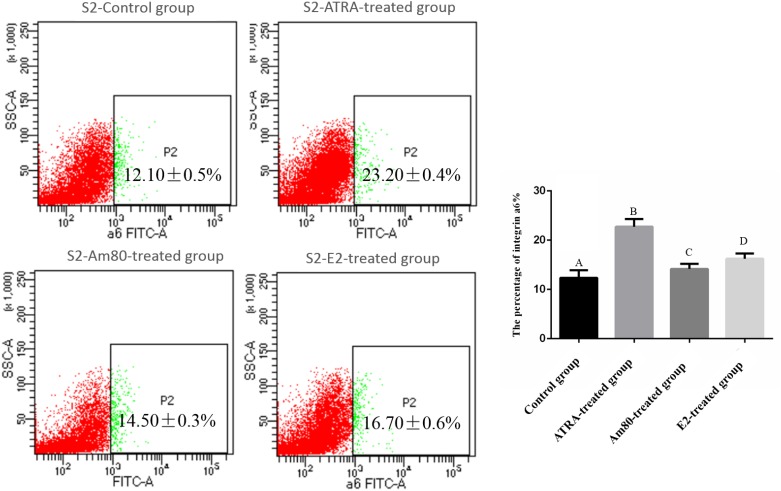
Detection of intergin α 6 positive cell by FACS. The results of FACS shows that the percentage of Control group is 12.10%; the percentage of ATRA-treated group is 23.20%; the percentage of Am80-treated group is 14.50%; the percentage of E2-treated group is 16.79%; A: Control group; B: ATRA-treated group; C: Am80-treated group; D: E2-treated group.

Expression of the surface antigens of SSEA-1 and Nanog were detected by cell chemoimmunology in embryoid bodies after a 4-day induction with ATRA, Am80, or E2, and in the control group. The results showed that positive clones of SSEA-1 and Nanog could be detected in four groups (Fig 4). SSEA-1 and Nanog are stem cell-specific markers and their expression may suggest that the cells still remained pluripotent. Integrin α6 and integrin β1 are SSC-specific genes. After a 10-day induction, integrin α6 surface antigens could be detected in the ATRA, but no or less in Am80, E2 groups and the control group. The number of positive clones was also different between the ATRA, Am80, and E2 groups. There were a higher number of positive clones in the ATRA than Am80 group and E2 group (Fig 5). The results of FACS showed that the percentage of ATRA-treated group is 23.20% (Fig 6).

## Discussion

Retinoic acid (RA) is the most active natural form of vitamin A, and its metabolites are 9-cis retinoic acid (9C-RA) and all-trans retinoic acid (ATRA). ATRA is the most common extracorporeal inducer of differentiation of various cells into germ cells. Cells treated with ATRA could change the expression or inhibition of specific genes, leading to changes in transcription factors, growth factors, extracellular matrix proteins, and so on. Accordingly, these changes could result in the differentiation, apoptosis, and signal transduction of cells, as shown by studies demonstrating that ATRA is necessary in the process of spermatogenesis in vivo and can promote PGC proliferation and survival in vitro[7,10,11]. ATRA-activated expression of germ cell-associated genes such as *Stra8*, *Sycp1*, and *Dazl*, induces mouse ESCs to differentiate into male germ cells. Am80 is an analog of retinoic acid that can specifically combine with RARα. Research has shown that the induction efficiency of Am80 is ten-fold that of retinoic acid[12]. Currently, Am80 is used primarily for the treatment of leukemia and related research, and the study of its induction of male germ cells in vitro has not been reported[13]. In birds, E2 is known to play a key role in the process of gonadal differentiation. The estrogen receptor (ERα) could be detected in female and male chicken gonads at 3.5 to 4.5 days after hatching. In one study, mouse embryonic stem cells (mESCs) transfected by pStra8-EGFP were induced by 1μg/mL E2 and co-cultured with mouse Sertoli cells. The researchers found that mESCs could form EBs and that E2 had certain inducing effects on differentiation of mouse ESCs into germ cells[21].

In order to establish a developmental model for germ cells in vitro and to select appropriate inducers for differentiating chicken ESCs into male germ cells, the current study used ATRA, Am80, and E2 to differentiate chicken ESCs toward the male germ cell lineage *in vitro*. we showed that ATRA, Am80, and E2 can cause expression of the *C-kit*, *Cvh*, *integrin α6*, and *integrin β1* genes, and accelerate the differentiation from ESCs to male germ cells. We also observed that after a 6-day induction period, ATRA is more effective in increasing the expression level of the *C-kit* and *Cvh* genes than Am80 or E2. Gene expression in the Am80-induced group did not change significantly in the earlier stage, but expression level was significantly increased after a 10-day induction period. This result is consistent with the results of Mi Meiling[22]. Pan Shaohui [21] used E2 to induce mouse ESCs to differentiate into the male germ cells in vitro and found that *Cvh* and other germ cell-specific gene expression levels increased significantly on the seventh day. In this study, expression of *Cvh* and *integrin β1* had no obvious increase in the E2-induced group until after the eighth day of induction, while the expression of *C-kit* and *integrin α6* had a rising trend in the process of induction. ATRA, Am80, and E2 can cause the expression of multiple reproductive cell-related genes, but the space-time dynamic expression of different genes is not consistent under the influence of different inducers. This may be related to the activation of different intracellular cytokines caused by the different inducers and different signal transduction processes, and the specific regulatory mechanisms still need further study.

Compared with the control group, positive clones of germ-like cells were detected by immunocytochemistry only in the ATRA induced groups. This indicates that ATRA can induce differentiation of chicken ESCs into male SSCs. In the ATRA-induced group, there were more germ-like cells gathered into clusters than in the other two induced groups, suggesting that ATRA may be a more appropriate inducer.

## Supporting Information

S1 Table The original data for Fig 3. (XLSX)
